# A294 CHOLESTASIS SECONDARY TO XANTHOGRANULOMATOUS CHOLANGITIS: A CASE REPORT

**DOI:** 10.1093/jcag/gwad061.294

**Published:** 2024-02-14

**Authors:** K A Labib, K Bishay

**Affiliations:** Gastroenterology, University of Alberta, Edmonton, AB, Canada; Gastroenterology, University of Alberta, Edmonton, AB, Canada

## Abstract

**Background:**

An 86-year-old female presented with four weeks of intermittent abdominal pain, postprandial nausea with an associated 10 lbs weight loss. Initial labs demonstrated a predominantly cholestatic liver enzyme elevation. Imaging revealed a thickened gallbladder with dilated CBD up to 15mm with no calculi, obstruction or pancreatic mass. EUS demonstrated a prominent ampulla with an area of hyperechogenicity, and ERCP was completed with sphincterotomy and biopsy of the ampulla. A cholangiogram showed a prominent common bile duct and a smooth distal taper without filling defects. Tumor markers, ampullary biopsy, and cytology of CBD brushings were negative for malignancy. Repeat ERCP for non-resolving symptoms with an extension of the sphincterotomy, brushings and insertion of a metal stent was completed without improvement in liver enzymes and a standard hepatitis workup returned negative. Her clinical course was further complicated by recurrent gram-negative bacteremia with CT findings of innumerable abscesses or metastasis in the liver. A US-guided targeted liver biopsy revealed large duct periductal tissue expansion by foamy macrophages with admixed neutrophils, eosinophils, plasma cells, and lymphocytes. These features were consistent with xanthogranulomatous cholangitis. We were planning to start ursodiol given the small duct disease however the patient succumbed to recurrent bacteremia and passed away.

**Aims:**

Review of methods to attain diagnosis of xanthogranulomatous cholangitis.

**Methods:**

Case Report.

**Results:**

Diagnosis of xanthogranulomatous cholangitis can be attained from targetted US-guided biopsy, surgical excision, or EUS-guided CBD biopsy.

**Conclusions:**

Xanthogranulomatous cholangitis is an extremely rare entity described in limited case reports, frequently mimicking neoplastic disease. Pathology is difficult to attain and historically case reports required surgical resections. One recent case reported diagnosis confirmed via EUS-guided FNA of the CBD. In our case, we attained the diagnosis through a targeted liver biopsy. Pathology of xanthogranulomatous cholangitis typically demonstrates foamy histiocytes, lipid-laden macrophages, eosinophils, lymphocytes, plasma cells, and fibrosis indicating xathogranulomas. The pathogenesis is thought to be an extension of xanthogranulomatous cholecystitis in which bile is extravasated into the gallbladder wall through Rokitansky-Aschoff sinuses or ulceration of the mucosa resulting in an inflammation caused by fibroblasts and macrophages as they phagocytose the lipids in bile, resulting in the formation of xanthoma cells. Given the rarity of the disease, there is no guideline on management and a majority of patients reported underwent surgical excision. This case demonstrates the utility of liver biopsy to establish this rare diagnosis, allowing for better detection and subsequently better-studied management.

**Funding Agencies:**

None

